# Serum albumin, prealbumin, and ischemia-modified albumin levels in patients with ANCA-associated vasculitis: A prospective cohort study

**DOI:** 10.1371/journal.pone.0271055

**Published:** 2022-07-07

**Authors:** Sung Soo Ahn, Taejun Yoon, Jason Jungsik Song, Yong-Beom Park, Sang-Won Lee

**Affiliations:** 1 Department of Internal Medicine, Yongin Severance Hospital, Yonsei University College of Medicine, Yongin, Republic of Korea; 2 Department of Medical Science, BK21 Plus Project, Yonsei University College of Medicine, Seoul, Republic of Korea; 3 Division of Rheumatology, Department of Internal Medicine, Yonsei University College of Medicine, Seoul, Republic of Korea; 4 Institute for Immunology and Immunological Diseases, Yonsei University College of Medicine, Seoul, Republic of Korea; East Carolina University Brody School of Medicine, UNITED STATES

## Abstract

**Objectives:**

Acute phase reactants (APRs) are proteins altered by inflammation and are regarded as surrogate markers representing inflammatory status. This study evaluated changes of albumin (Alb), prealbumin (Palb), and ischemia-modified albumin (IMA) in patients with anti-neutrophil cytoplasmic antibody (ANCA)-associated vasculitis (AAV) in response to alterations in disease activity and the correlation between disease activity and Alb, Palb, and IMA.

**Methods:**

Fifty-nine patients with AAV registered in the prospective SHAVE cohort, who had available serial blood samples at least three months apart were included (indicated as pre and post). Correlation analysis and linear regression were carried out to determine the relationship between continuous variables. Alb, Palb, and IMA levels in 40 healthy controls (HCs) were compared with patients with AAV.

**Results:**

Comparison of Alb, Palb, and IMA levels in HCs and in patients at initial (pre) and follow-up (post) time points revealed that Alb levels significantly increased following the improvement of disease activity and were comparable between HCs and patients at follow-up (post). Meanwhile, there was no significant difference noted in Palb and IMA levels after the decrease of disease activity. While initial (pre) Alb and Palb were significantly associated with BVAS, a subgroup analysis of patients with new-onset disease showed Palb was no longer significantly associated with Birmingham Vasculitis Activity Score (BVAS). Multivariate linear regression showed Alb level (standardized β = -0.377; 95% confidence interval: -5.623, -1.260; p = 0.003) was an independent predictor of BVAS at baseline.

**Conclusions:**

Among Alb, Palb, and IMA, we found that Alb could be a useful marker indicating disease activity in patients with AAV.

## Introduction

Anti-neutrophil cytoplasmic antibody (ANCA)-associated vasculitis (AAV), a disease entity that includes microscopic polyangiitis (MPA), granulomatosis with polyangiitis (GPA), and eosinophilic granulomatosis with polyangiitis (EGPA), causes injury to small vessels that results in necrotizing vascular inflammation [1]. While substantial advances have been made in the management of AAV, it remains a potentially life- and organ-threatening disorder [2]. Accurate assessment of disease activity is imperative in the management of AAV, as it not only affects the physicians’ choice of therapy but also influences subsequent adjustment of medications [3]. Among measures developed to evaluate disease activity in AAV, the third version of the Birmingham Vasculitis Activity Score (BVAS 3.0) has been adopted as a reliable measure that reflects global and localized inflammation most comprehensively [4]. However, the estimation of BVAS is complex, requires a considerable amount of time, and could be subject to inter-observer bias among investigators. Thus, there remains a substantial need to develop objective laboratory indices that are easy to use and could successfully reflect inflammation in AAV.

Acute phase reactants (APRs) are defined as proteins altered by the onset of inflammation and are regarded as surrogate markers representing inflammatory status [5]. APRs are divided into positive and negative APRs according to their direction of change in response to inflammation. In the clinical setting, C-reactive protein (CRP) and erythrocyte sedimentation rate (ESR) are the most widely utilized positive APRs as they increase in the presence of inflammation. In contrast, albumin (Alb) and prealbumin (Palb) are considered negative APRs because they decrease in response to the severity of inflammation. Alb, primarily produced in the liver, is the most abundant form of protein found in the human blood and is responsible for regulating various physiologic activities [6]. Increased inflammation contributes to the decrease of Alb by affecting its synthesis, catabolism, and extracellular/intracellular distribution. However, as the half-life of Alb is reported to be approximately 20 days, the clinical utility of serum Alb levels in assessing rapid changes of inflammatory response is limited. Meanwhile, Palb, a precursor of Alb, has a short half-life of 2–4 days. It has been suggested as a sensitive and cost-effective laboratory test to assess the illness severity and correlates well with patient outcomes [7]. In addition, ischemia-modified Alb (IMA) is a type of Alb with an altered structural conformation of the N-terminus of Alb that increases upon exposure to ischemia, hypoxia, oxidative stress, and inflammation [8]. Although IMA was initially recognized as a biological marker increased in acute coronary syndrome, research has demonstrated that IMA is also elevated in patients with autoimmune disorders such as inflammatory bowel diseases, Behcet disease, and ankylosing spondylitis [9–11]. Nonetheless, the correlation between disease activity and indices of Alb, Palb, and IMA has not been investigated in patients with AAV. Therefore, the objective of this study was to assess the changes in serum Alb, Palb, and IMA levels in patients with AAV in response to alterations in AAV disease activity.

## Methods

### Patient cohort, demographics, and clinical data

The Severance Hospital ANCA-associated VasculitidEs (SHAVE) cohort was a prospective cohort that consisted of patients with AAV (MPA, GPA, and EGPA). Patients were subjected to clinical and laboratory data assessment and had their blood obtained during routine patient care [12]. After obtaining patient blood, sera were separated immediately from the whole blood by centrifugation and stored in a deep freezer at -70°C before usage. Patients who fulfilled the criteria for AAV following the algorithm proposed by the 2007 European Medicine Agency and the definitions set by the 2012 Chapel Hill Consensus Conference were included [13, 14]. In addition, patients with infection and autoimmune disease other than AAV were excluded. This study included a total of 59 patients with AAV registered in the SHAVE cohort between November 2016 and July 2020, and had available serial blood samples at least three months apart (indicated as pre and post). Included patients demonstrated a disease activity that decreased or remained stable on follow-up (post) compared to baseline (pre).

As for the demographics and baseline clinical data of the patients, age, sex, disease duration, and patient diagnosis were collected. Regarding AAV-related variables, BVAS (version 3), five-factor score (FFS) 2009, and short form-36 (SF-36) were assessed [15–17]. New-onset AAV was defined as patients with a disease duration of less than one month, and the organ involvement of patients was assigned according to the clinical features comprising the BVAS version 3. The immunosuppressive agents prescribed to the patients were also investigated. All enrolled patients provided written informed consent for blood sample collection. The Institutional Review Board of Severance Hospital approved this study (4-2016-0901), and relevant study procedures were performed in accordance with the principles set forth in the 1964 Helsinki Declaration and its later amendments and comparable standards.

### Laboratory results and further tests using stored patient sera

Along with the obtained clinical data, the following routine laboratory tests were retrieved: white blood cell (WBC) count, hemoglobin, lymphocyte count, creatinine, aspartate aminotransferase, alanine aminotransferase, Alb, ESR, and CRP. Additionally, the results of ANCA serology (perinuclear- [p-ANCA] or myeloperoxidase-ANCA [MPO-ANCA], cytoplasmic- or proteinase 3-ANCA, and ANCA-negative) were also collected. Palb (Abcam, Cambridge, UK) and IMA (Cusabio Biotech, Wuhan, China) levels in patient sera were estimated by using commercial enzyme-linked immunosorbent assay kits. For comparison with patients with AAV, serum levels of Palb and IMA were also evaluated in 40 healthy controls (HCs).

### Statistical analyses

Statistical analyses were conducted using the MedCalc statistical software version 20 (MedCalc Software Ltd., Ostend, Belgium). Continuous data were presented as median (interquartile ranges), whereas categorical variables were presented as number (percentages). Statistical differences between continuous variables were assessed using the Kruskal-Wallis test and the Mann-Whitney U test. Pearson’s correlation analysis was carried out to determine the relationship between continuous variables, and multivariate linear regression analysis with a forward entry method was done using variables that showed significance in univariate analysis to identify predictive laboratory tests for BVAS. For all statistical analyses, a two-tailed p-value of <0.05 was considered significant.

## Results

### Baseline patient characteristics

Baseline clinical and laboratory characteristics of the patients are shown in Table 1. The median age was 64.0 years, and the median disease duration was 0.0 months. Of 59 patients, 38 (64.4%) were female. The diagnosis of MPA accounted for nearly half (49.2%) of the patients, followed by GPA (30.5%) and EGPA (20.3%). The median BVAS, FFS, SF-36 physical component score (PCS), and SF-36 mental component score (MCS) were 12.0, 0.0, 50.6, and 54.1, respectively. For organ involvement, chest involvement was most frequently observed. Additionally, p-ANCA and MPO-ANCA were the most commonly detected ANCA types (Table 1).

**Table 1:** Baseline clinical data and laboratory characteristics of patients.

	Total number of patients (n = 59)
**Clinical data**	
**Demographics**	
Age, years	64.0 (51.3–72.0)
Disease duration, months	0.0 (0.0–5.0)
Female sex	38 (64.4)
**Diagnosis**	
MPA	29 (49.2)
GPA	18 (30.5)
EGPA	12 (20.3)
**AAV-related variables**	
BVAS version 3	12.0 (6.0–17.8)
FFS (2009)	0.0 (0.0–2.0)
Short form-36 PCS	50.6 (30.8–68.0)
Short form-36 MCS	54.1 (35.2–71.2)
**Pattern of organ involvement**	
General	27 (45.8)
Cutaneous	10 (16.9)
Mucous membranes/eyes	2 (3.4)
Ear, nose, and throat	25 (42.4)
Chest	44 (74.6)
Cardiovascular	5 (8.5)
Abdominal	2 (3.4)
Renal	34 (57.6)
Nervous system	20 (33.9)
**Treatment**	
Glucocorticoids	45 (76.3)
Cyclophosphamide	6 (10.2)
Rituximab	3 (5.1)
Azathioprine	16 (27.1)
Methotrexate	1 (1.7)
**Laboratory results**	
**Laboratory tests**	
WBC count (/mm^3^)	8400.0 (5610.0–12795.0)
Lymphocyte count (/mm^3^)	1410.0 (882.5–1775.0)
Creatinine (mg/dL)	0.9 (0.6–2.0)
Aspartate aminotransferase (IU/L)	19.0 (14.0–22.0)
Alanine aminotransferase (IU/L)	19.0 (11.3–24.8)
Albumin (g/dL)	3.6 (3.0–4.1)
ESR (mm/hr)	30.0 (9.0–82.5)
CRP (mg/L)	5.2 (1.1–39.6)
**ANCA serology**	
p- or MPO-ANCA	39 (66.1)
c- or PR3-ANCA	6 (10.2)
ANCA-negative	14 (23.7)

Continuous variables are presented as median (interquartile range) while categorical variables are presented as number (percentage).

MPA: microscopic polyangiitis; GPA: granulomatosis with polyangiitis; EGPA: eosinophilic granulomatosis with polyangiitis; AAV: anti-neutrophil cytoplasmic antibody-associated vasculitis; BVAS: Birmingham Vasculitis Activity Score; FFS: five-factor score; PCS: physical component score; MCS: mental component score; WBC: white blood cell; ESR: erythrocyte sedimentation rate; CRP: C-reactive protein; ANCA: anti-neutrophil cytoplasmic antibody; P: perinuclear; MPO: myeloperoxidase; C: cytoplasmic; PR3: proteinase 3.

**Fig 1:**
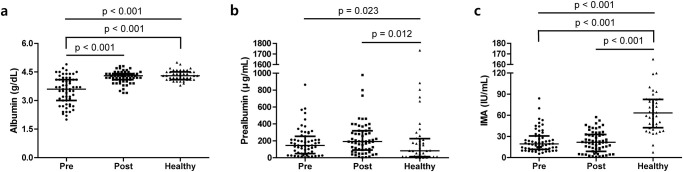
Albumin, prealbumin, and ischemia-modified albumin levels upon serial testing and comparison with healthy controls. (a) Albumin levels were significantly lower at baseline compared with levels at follow-up and in healthy controls. (b) Prealbumin was found to be significantly higher at follow-up than in healthy controls. (c) Ischemia-modified albumin levels were significantly higher in healthy controls than the levels at baseline and at follow-up. The error bars present median and interquartile range. IMA: ischemia-modified albumin.

Initial levels of the median Alb, Palb, and IMA of the patients were 3.6 g/dL, 145.5 μg/mL, and 19.4 IU/mL, respectively. Comparison of Alb, Palb, and IMA levels in HCs and in patients at initial (pre) and follow-up (post) time points revealed that Alb levels significantly increased following the improvement of disease activity and were comparable between HCs and patients at follow-up (post) (Fig 1a). Meanwhile, there was no significant difference noted in Palb and IMA levels after the decrease of disease activity. However, Palb was significantly higher, and IMA was significantly lower at follow-up (post) than HCs (Fig 1b and 1c).

### Correlation of Alb, Palb, and IMA with AAV-related variables at baseline

Alb was found to be significantly associated with all AAV-related variables and showed a strong correlation with BVAS (r = -0.664; 95% confidence interval [CI]: -0.787, -0.492; p<0.001) and SF-36 PCS (r = 0.614; 95% CI: 0.425, 0.752; p<0.001). In addition, Alb was correlated with WBC and lymphocyte count, ESR, and CRP level. Similarly, Palb was found to be significantly associated with AAV-related variables, but the correlation coefficient with BVAS and SF-36 PCS was lower compared with that with Alb. Palb was only correlated with the laboratory findings of ESR and CRP. On the other hand, IMA did not show a significant correlation with AAV-related variables. Furthermore, there was no association between IMA and the laboratory tests studied, except WBC count (r = 0.285, 95% CI 0.031, 0.504, p = 0.029) (Table 2).

**Table 2:** Association of albumin, prealbumin, and ischemia-modified albumin with AAV-related variables and laboratory tests at baseline.

	Albumin			Prealbumin			Ischemia-modified albumin		
	Correlation coefficient	95% CI	p-value	Correlation coefficient	95% CI	p-value	Correlation coefficient	95% CI	p-value
**AAV-related variables**									
BVAS version 3	-0.664	-0.787, -0.492	<0.001	-0.304	-0.520, -0.052	0.019	0.093	-0.167, 0.341	0.485
FFS (2009)	-0.287	-0.506, -0.033	0.028	-0.323	-0.535, -0.073	0.013	-0.025	-0.279, 0.233	0.851
Short form-36 PCS	0.614	0.425, 0.752	<0.001	0.350	0.103, 0.556	0.007	-0.076	-0.325, 0.184	0.570
Short form-36 MCS	0.499	0.278, 0.669	<0.001	0.394	0.153, 0.591	0.002	-0.147	-0.389, 0.113	0.267
**Laboratory tests**									
WBC count	-0.310	-0.524, -0.058	0.017	-0.137	-0.380, 0.124	0.302	0.285	0.031, 0.504	0.029
Lymphocyte count	0.334	0.085, 0.544	0.010	-0.063	-0.314, 0.196	0.633	0.069	-0.191, 0.319	0.604
Creatinine	0.079	-0.181, 0.329	0.551	-0.136	-0.379, 0.125	0.306	0.012	-0.245, 0.267	0.927
Aspartate aminotransferase	-0.035	-0.289, 0.223	0.792	-0.183	-0.420, 0.077	0.165	0.036	-0.222, 0.290	0.786
Alanine aminotransferase	-0.143	-0.385, 0.117	0.280	0.029	-0.229, 0.283	0.828	0.073	-0.187, 0.323	0.585
ESR	-0.555	-0.710, -0.349	<0.001	-0.333	-0.542, -0.084	0.010	-0.010	-0.265, 0.247	0.943
CRP	-0.667	-0.789, -0.496	<0.001	-0.424	-0.613, -0.188	<0.001	-0.075	-0.325, 0.184	0.571

Data are shown as correlation coefficient (p-value).

CI: confidence interval; AAV: anti-neutrophil cytoplasmic antibody-associated vasculitis; BVAS: Birmingham Vasculitis Activity Score; FFS: five-factor score; PCS: physical component score; MCS: mental component score; WBC: white blood cell; ESR: erythrocyte sedimentation rate; CRP: C-reactive protein.

When a subgroup analysis of patients with new-onset disease (n = 37) was performed, Alb was observed to be associated with BVAS (r = -0.434, 95% CI -0.665, -0.128, p = 0.007), whereas Palb was no longer significantly associated (r = 0.259, 95% CI -0.538, 0.071, p = 0.122). However, both Alb (r = -0.669, 95% CI -0.816, -0.440, p<0.001) and Palb (r = -0.534, 95% CI -0.731, -0.253, p<0.001) were found to have a significant correlation with CRP level (Table 3).

**Table 3:** Association of albumin, prealbumin, and ischemia-modified albumin with AAV-related variables and laboratory tests in new-onset AAV at baseline (n = 37).

	Albumin			Prealbumin			Ischemia-modified albumin		
	Correlation coefficient	95% CI	p-value	Correlation coefficient	95% CI	p-value	Correlation coefficient	95% CI	p-value
**AAV-related variables**									
BVAS version 3	-0.434	-0.665, -0.128	0.007	-0.259	-0.538, 0.071	0.122	-0.126	-0.432, 0.207	0.459
FFS (2009)	-0.164	-0.463, 0.169	0.333	-0.377	-0.625, -0.060	0.021	-0.259	-0.538, 0.071	0.121
Short form-36 PCS	0.402	0.090, 0.643	0.014	0.324	-0.000, 0.586	0.051	0.119	-0.213, 0.427	0.483
Short form-36 MCS	0.392	0.078, 0.635	0.016	0.377	0.061, 0.625	0.021	0.004	-0.321, 0.327	0.983
**Laboratory tests**									
WBC count	-0.159	-0.459, 0.174	0.348	-0.000	-0.324, 0.324	0.999	0.237	-0.095, 0.521	0.159
Lymphocyte count	0.273	-0.056, 0.548	0.102	-0.174	-0.472, 0.159	0.303	0.140	-0.193, 0.444	0.408
Creatinine	-0.150	-0.452, 0.183	0.377	-0.047	-0.366, 0.281	0.780	-0.275	-0.550, 0.053	0.099
Aspartate aminotransferase	-0.164	-0.463, 0.169	0.332	-0.279	-0.553, 0.049	0.094	-0.023	-0.345, 0.303	0.890
Alanine aminotransferase	-0.173	-0.470, 0.160	0.307	-0.054	-0.371, 0.275	0.753	0.073	-0.257, 0.388	0.666
ESR	-0.360	-0.613, -0.041	0.029	-0.321	-0.584, 0.003	0.053	-0.219	-0.507, 0.114	0.194
CRP	-0.669	-0.816, -0.440	<0.001	-0.534	-0.731, -0.253	<0.001	-0.227	-0.513, 0.105	0.177

Data are shown as correlation coefficient (p-value).

CI: confidence interval; AAV: Anti-neutrophil cytoplasmic antibody-associated vasculitis; BVAS: Birmingham Vasculitis Activity Score; FFS: five-factor score; PCS: physical component score; MCS: mental component score; WBC: white blood cell; ESR: erythrocyte sedimentation rate; CRP: C-reactive protein.

**Fig 2:**
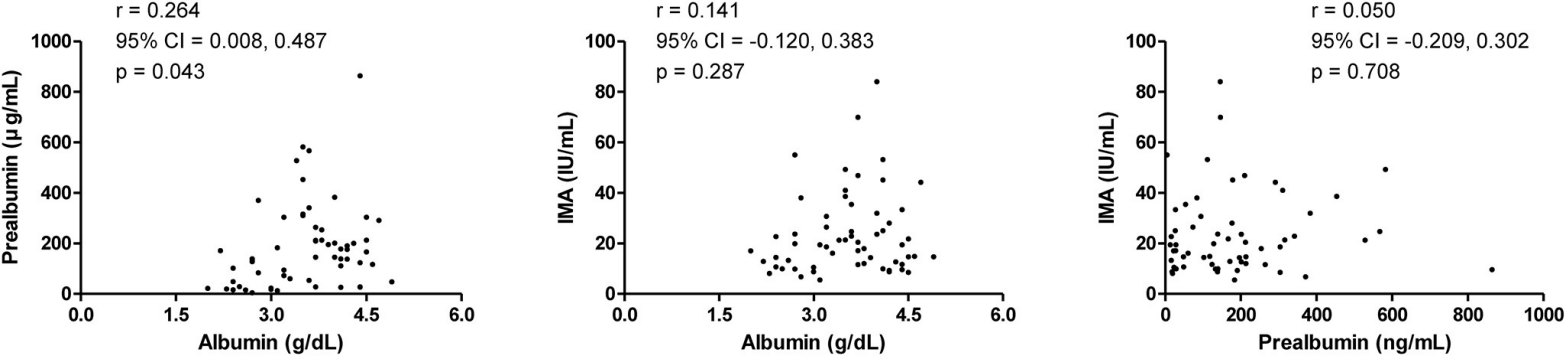
Relationship among baseline albumin, prealbumin, and ischemia-modified albumin. A significant association was found between albumin and prealbumin in patients with ANCA-associated vasculitis. ANCA: anti-neutrophil cytoplasmic antibody; IMA: ischemia-modified albumin.

Regarding the correlation among Alb, Palb, and IMA, Alb was significantly correlated with Palb (r = 0.264, 95% CI 0.008, 0.487, p = 0.043). However, the associations between Alb and IMA, and Palb and IMA were not evident (Fig 2).

### Association between laboratory tests and BVAS at baseline

In univariate analysis, WBC count, lymphocyte count, Alb level, ESR, and CRP level were significantly associated with BVAS. However, multivariate analysis showed that lymphocyte count (standardized β = -0.244; 95% CI: -0.4224, -0.438; p = 0.017), Alb level (standardized β = -0.377; 95% CI: -5.623, -1.260; p = 0.003), and ESR (standardized β = -0.371; 95% CI: -0.023, 0.095; p = 0.002) were independently predictive of BVAS (Table 4).

**Table 4:** Univariate and multivariate linear regression between laboratory tests and BVAS version 3 at baseline.

Laboratory tests	Univariate analysis			Multivariate analysis		
	Beta	95% CI	p-value	Beta	95% CI	p-value
WBC count	0.271	0.020, 0.657	0.038			
Lymphocyte count	-0.364	-5.824, -1.112	0.005	-0.244	-4.224, -0.438	0.017
Creatinine	0.003	-0.536, 0.547	0.983			
Aspartate aminotransferase	-0.045	-0.294, 0.209	0.735			
Alanine aminotransferase	0.015	-0.126, 0.141	0.912			
Albumin	-0.664	-7.885, -4.260	<0.001	-0.377	-5.623, -1.260	0.003
ESR	0.576	0.057, 0.126	<0.001	0.371	0.023, 0.095	0.002
CRP	0.416	0.023, 0.088	0.001			

CI: confidence interval; BVAS: Birmingham Vasculitis Activity Score; WBC: white blood cell; ESR: erythrocyte sedimentation rate; CRP: C-reactive protein.

### Effect of ANCA serology, renal involvement, medications, diagnosis, and disease duration on baseline Alb, Palb, and IMA levels

**Fig 3:**
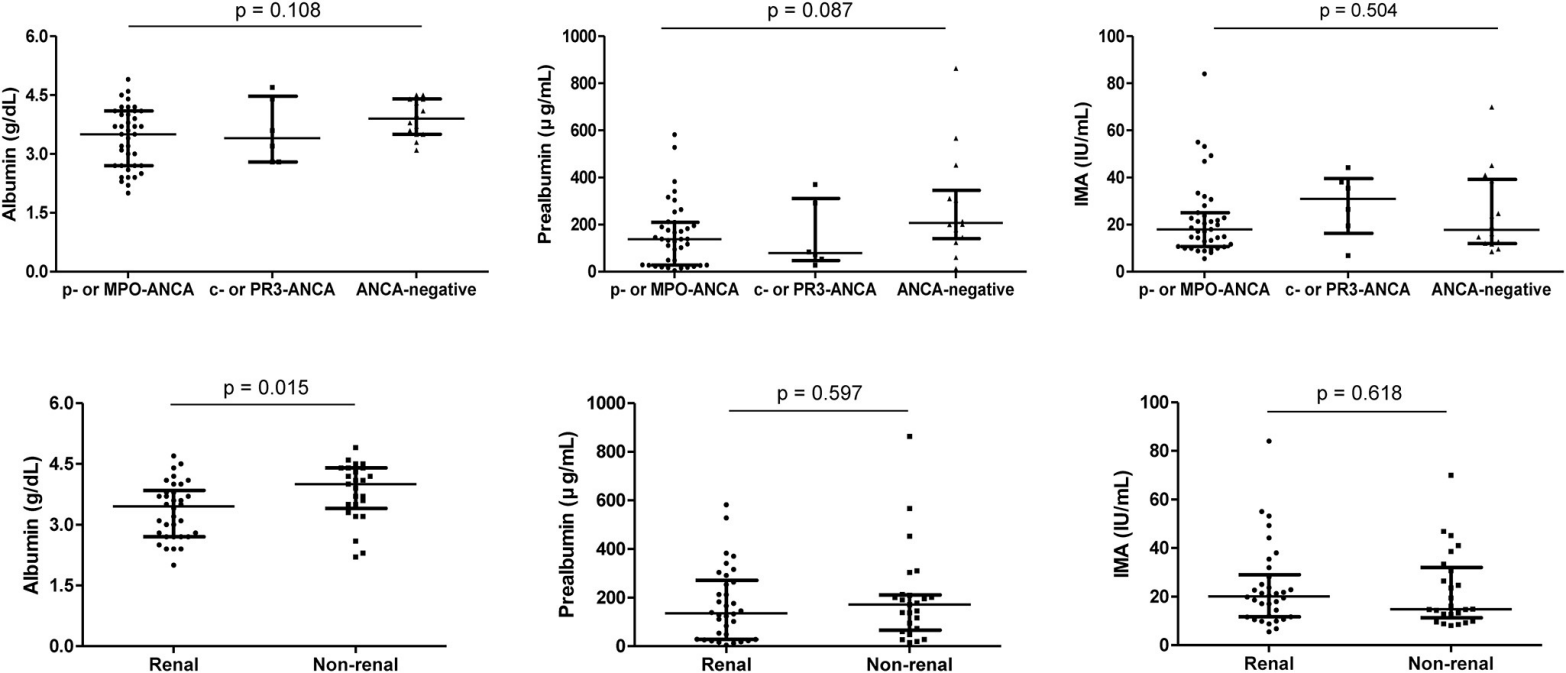
Baseline albumin, prealbumin, and ischemia-modified albumin levels based on ANCA serology and renal involvement. ANCA serology did not significantly affect levels of albumin, prealbumin, and ischemia-modified albumin. Meanwhile, albumin level was significantly lower in patients with renal involvement that in those without. The error bars present median and interquartile range. ANCA: anti-neutrophil cytoplasmic antibody; P: perinuclear; MPO: myeloperoxidase; C: cytoplasmic; PR3: proteinase 3; IMA: ischemia-modified albumin.

**Fig 4:**
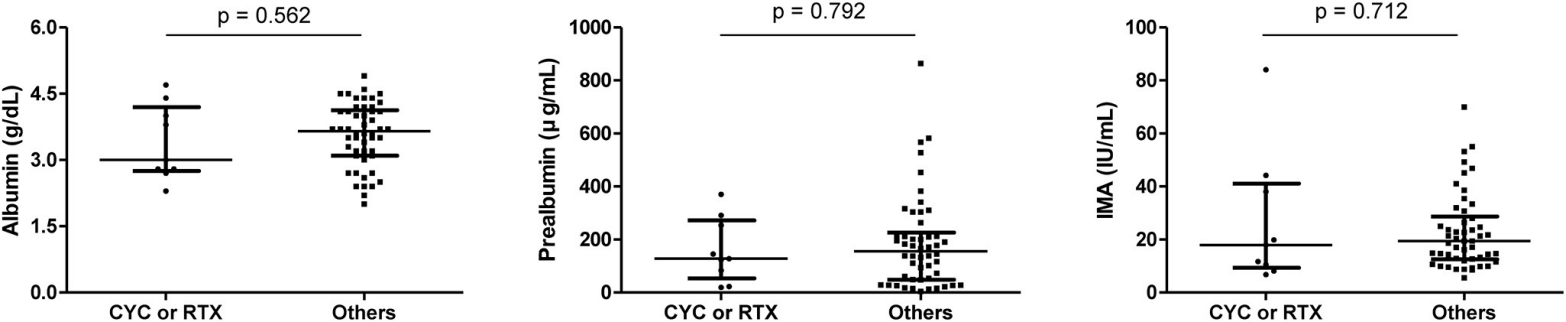
Comparison of baseline albumin, prealbumin, and ischemia-modified albumin levels according to treatment. Albumin, prealbumin, and ischemia-modified albumin levels were not significantly different in patients with concurrent treatment with cyclophosphamide or rituximab and in those without. The error bars present median and interquartile range. CYC: cyclophosphamide; RTX: rituximab; IMA: ischemia-modified albumin.

**Fig 5:**
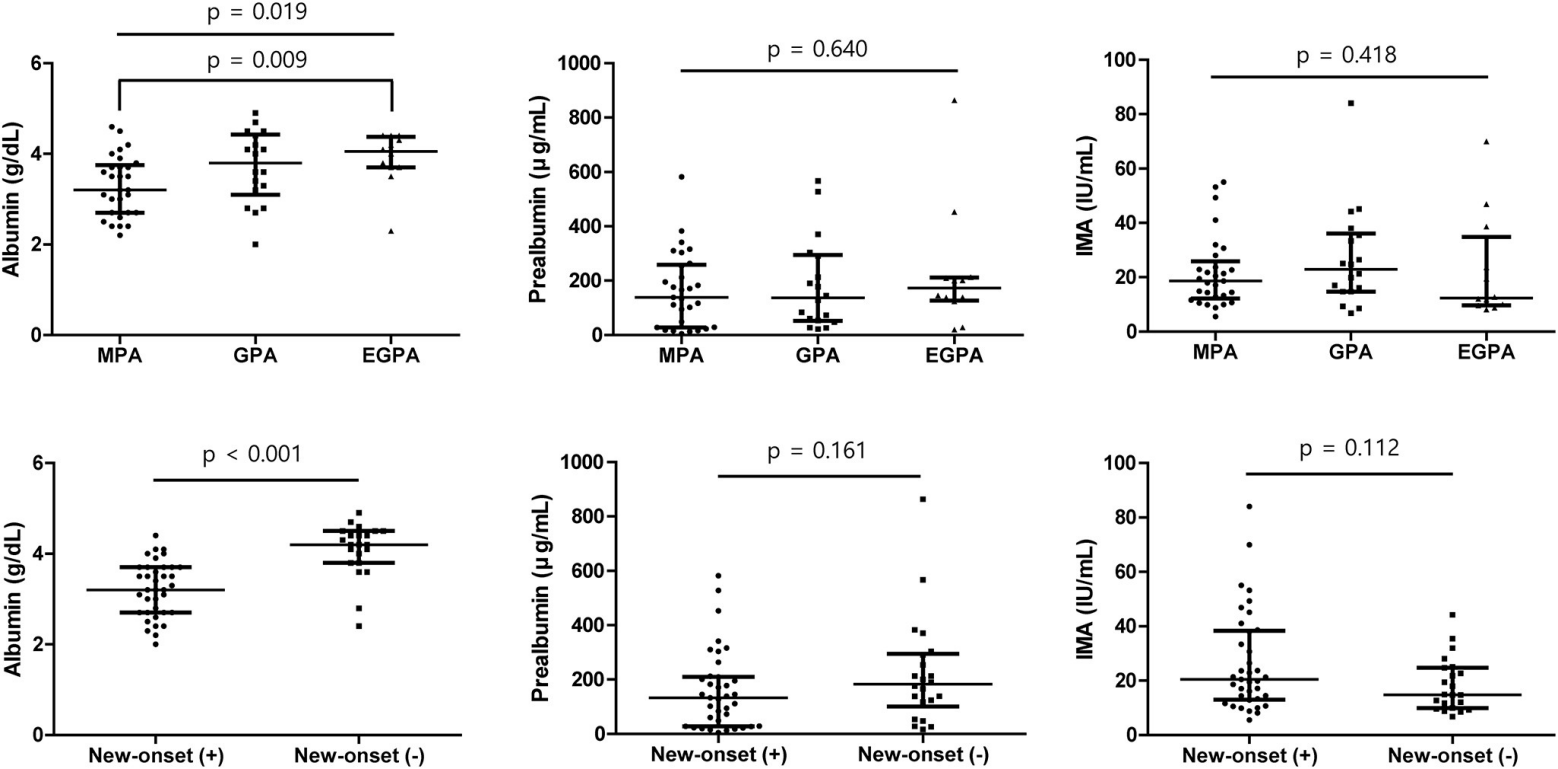
Levels of initial albumin, prealbumin, and ischemia-modified albumin based on disease diagnosis and new-onset disease. Patients with MPA had significantly lower albumin level compared to those with EGPA, but prealbumin and ischemia-modified albumin levels were comparable in patients with MPA, GPA, and EGPA. Among patients with and without new-onset disease, a significantly decreased albumin level was observed in those with new-onset disease, while no difference was noted regarding prealbumin and ischemia-modified albumin levels. The error bars present median and interquartile range. MPA: microscopic polyangiitis; EGPA: eosinophilic granulomatosis with polyangiitis GPA: granulomatosis with polyangiitis.

Comparing the levels of Alb, Palb, and IMA in patients, the ANCA serology did not significantly affect the levels of Alb, Palb, and IMA. In contrast, Alb level was significantly lower in patients with renal involvement than in those without (p = 0.015) (Fig 3). Alb, Palb, and IMA levels were also comparable between patients who are currently on cyclophosphamide or rituximab treatment and patients without such treatment (Fig 4). Concerning disease diagnosis, Alb level revealed to be significantly lower in patients with MPA compared to patients with EGPA (p = 0.009), but there was no difference in Palb and IMA levels according to diagnosis. In those with and without new-onset disease, a significantly lower Alb level was noted in those with new-onset disease (p<0.001), but Palb and IMA levels did not differ (Fig 5).

## Discussion

In this study, we assessed the relationship of Alb, Palb, and IMA with disease activity in AAV. Alb and Palb were revealed to be significantly associated with the AAV variables BVAS, FFS, and SF-36. Further, Alb level was observed to increase in patients after treatment and was independently associated with BVAS. Importantly, Alb, Palb, and IMA levels after treatment was unaffected by ANCA serotypes and therapeutic medications. Finally, Alb level was found to be significantly lower in patients with renal manifestation, one of the life-threatening complications of AAV. Taken together, our results suggest that Alb could be a feasible marker of AAV disease activity and has utility in predicting kidney involvement.

Among Alb, Palb, and IMA, we demonstrated that Alb had a high correlation with disease activity at baseline, and the correlation was consistent in those with new-onset disease. Moreover, the correlation coefficient of Alb with BVAS was over two times higher than that of Palb. Since the inflammatory cytokines TNF-α, IL-1, IL-6, and IL-8 play an essential role in the pathogenesis of AAV and negatively impact Alb levels, it could be suggested that hypoalbuminemia present in patients with AAV are proportional to the degree of inflammation [18, 19]. Nevertheless, the initial Palb level (pre) was comparable between patients with AAV and HCs. Additionally, Palb level in the follow-up (post) time point was significantly higher than HCs, and Palb was not associated with disease activity in the subgroup of new-onset disease. Considering that Palb is generally considered to have a more rapid turnover than Alb and is generally thought to decrease as a consequence of systemic inflammation, this finding could be somewhat unexpected. However, this finding could be attributable to the fact that Palb is chiefly affected by protein ingestion and nutritional state and could even decrease during intensive stress [7, 20], and the increased level of Palb in the follow-up (post) than in healthy controls might be a reflection of a compensatory mechanism [21]. In this context, it has been recommended that the Palb should be interpreted with caution in a clinical setting where acute inflammation is present [22]. Likewise, IMA did not correlate with BVAS at baseline in our study population or in those with new-onset disease. Furthermore, in contrast with previous literature [23], IMA was not associated with Alb and was higher in HCs than in patients with AAV both in the initial (pre) and follow-up (post) period, which may be partly explained by the difference in the disease being investigated and the microenvironment of the affected tissues. Collectively, the results of our study suggest that Alb, but not Palb and IMA, is related to the disease activity of AAV.

Alb is a laboratory test that is routinely measured in general clinical practice. While it is traditionally regarded that Alb is associated with the nutritional status of an individual, Alb actually has a relatively low sensitivity and specificity in measuring changes in nutrition and is substantially affected by heightened inflammation [24]. Studies have reported that the decrease in Alb is suitable for assessing the severity of disease in rheumatoid arthritis, systemic lupus erythematosus, and ulcerative colitis [25–28]. Interestingly, the observations from this study confirmed the previously obtained data of our group, which showed hypoalbuminemia at AAV diagnosis was associated with disease activity and lymphocyte count [29]. Furthermore, the correlation coefficient between BVAS and Alb (BVAS vs. Alb: r = -0.664; 95% CI: -0.787, -0.492; p<0.001) was higher than that of BVAS with ESR (BVAS vs. ESR: r = 0.576; 95% CI: 0.375, 0.725; p<0.001) and BVAS with CRP (BVAS vs. CRP: r = 0.416; 95% CI: 0.180, 0.608; p = 0.001). Furthermore, serial laboratory testing of Alb confirmed its clinical utility as a potential marker of monitoring disease activity. Additionally, it is advantageous that Alb testing is readily available and could be assessed conveniently and at a low cost with a high reproducibility. Thus, it has great utility as a serologic marker that reflects changes in clinical activity.

The main strength of this study is that paired samples of patients were utilized to investigate the dynamic changes of Alb, Palb, and IMA, along with disease severity. Nevertheless, several limitations should also be addressed. First, the number of patients included in this study was small, which prevented us from performing robust subgroup analysis. Second, the prognostic implication of Alb could not be assessed due to the limited number of patients. Third, given that the Alb level is reported to be influenced by multiple factors besides inflammation, further studies addressing this concern are needed to validate the findings of our study.

## Conclusions

Among Alb, Palb, and IMA, we found that Alb and Palb were associated with disease activity in patients with AAV. Alb levels significantly increased after treatment, suggesting that it could be a useful marker indicating disease activity. Larger studies are required to verify whether Alb could be a reliable indicator of disease activity in AAV.

## Supporting information

S1 File Dataset that was used for analysis. (XLSX)

## Abbreviations

AAV: anti-neutrophil cytoplasmic antibody-associated vasculitis
Alb: albumin
ANCA: anti-neutrophil cytoplasmic antibody
APR: acute phase reactant
BVAS: Birmingham Vasculitis Activity Score
CI: confidence interval
CRP: C-reactive protein
EGPA: eosinophilic granulomatosis with polyangiitis
ESR: erythrocyte sedimentation rate
FFS: five-factor score
GPA: granulomatosis with polyangiitis
HC: healthy control
IMA: ischemia-modified albumin
MCS: mental component score
MPA: microscopic polyangiitis
MPO: myeloperoxidase
P: perinuclear
Palb: prealbumin
PCS: physical component score
SHAVE: Severance Hospital ANCA-associated VasculitidEs
WBC: white blood cell
